# Circulating Ligands of the Receptor for Advanced Glycation End Products and the Soluble Form of the Receptor Modulate Cardiovascular Cell Apoptosis in Diabetes

**DOI:** 10.3390/molecules25225235

**Published:** 2020-11-10

**Authors:** James N. Tsoporis, Erifili Hatziagelaki, Sahil Gupta, Shehla Izhar, Vasileos Salpeas, Anastasia Tsiavou, Angelos G. Rigopoulos, Andreas S. Triantafyllis, John C. Marshall, Thomas G. Parker, Ioannis K. Rizos

**Affiliations:** 1 Keenan Research Centre for Biomedical Science, Li Ka Shing Knowledge Institute, Unity Health Toronto, University of Toronto, Toronto, ON M5B 1T8, Canada; Sahil.Gupta@unityhealth.to (S.G.); Shehla.Izhar@unityhealth.to (S.I.); john.marshall@unityhealth.to (J.C.M.); thomas.parker@unityhealth.to (T.G.P.); 2 Department of Internal Medicine, Research Institute and Diabetes Center, Attikon University Hospital, University of Athens Medical School, 124 62 Athens, Greece; erihat@med.uoa.gr (E.H.); anatsiav@yahoo.com (A.T.); 3 Institute of Medical Science, University of Toronto, Toronto, ON M5S 1A6, Canada; 4 Academic Department of Cardiology, Attikon University Hospital, University of Athens Medical School, 12462 Athens, Greece; vsalpeas@med.uoa.gr (V.S.); angelos.rigopoulos@gmail.com (A.G.R.); andreas.triantafyllis@gmail.com (A.S.T.); ioannis.c.rizos@otenet.gr (I.K.R.)

**Keywords:** S100 proteins, diabetes, receptor for advanced glycation end products, apoptosis

## Abstract

We determined whether plasma concentrations of the receptor for advanced glycation end products (RAGE) and the soluble (s) form of RAGE (sRAGE) in healthy individuals and patients with type 2 diabetes (T2D) modulate vascular remodeling. Healthy individuals and patients with T2D were divided into two age groups: young = <35 years old or middle-aged (36–64 years old) and stratified based on normal glucose tolerance (NGT), impaired (IGT), and T2D. Plasma titers of sRAGE, the RAGE ligands, AGEs, S100B, S100A1, S100A6, and the apoptotic marker Fas ligand Fas(L) were measured by enzyme-linked immunosorbent assay (ELISA). The apoptotic potential of the above RAGE ligands and sRAGE were assessed in cultured adult rat aortic smooth muscle cells (ASMC). In NGT individuals, aging increased the circulating levels of AGEs and S100B and decreased sRAGE, S100A1 and S100A6. Middle-aged patients with T2D presented higher levels of circulating S100B, AGEs and FasL, but lower levels of sRAGE, S100A1 and S100A6 than individuals with NGT or IGT. Treatment of ASMC with either AGEs or S100B at concentrations detected in T2D patients increased markers of inflammation and apoptosis. Responses attenuated by concomitant administration of sRAGE. In middle-aged patients with T2D, lower circulating plasma levels of sRAGE may limit decoy and exogenous trapping of deleterious pro-apoptotic/pro-inflammatory RAGE ligands AGEs and S100B, increasing the risk for diabetic complications.

## 1. Introduction

Diabetes mellitus can result from insulin deficiency (Type 1-T1D) or insulin resistance (Type 2-T2D) and constitutes a worldwide epidemic [1,2,3]. Several biological changes influence the onset and pathogenesis of diabetes, but the accumulation of advanced glycation end products is indispensable for the disease [4,5]. AGEs exert their effects in several ways, including modulation of the receptor for advanced glycation end products (RAGE) [6,7,8]. Evidence in the literature supports the hypothesis that AGEs and non-AGEs ligands of the RAGE accumulate in the plasma/serum of human subjects with diabetes, potentially serving as unreported biomarkers for diabetes-induced complications [6,7,8]. RAGE is a pattern recognition receptor that recognizes diverse ligands, including AGEs, some S100/calgranulins, amyloid-beta peptide, and High Mobility Group Box 1 protein (HMGB1), all of which are collectively called damage-associated molecular patterns (DAMPS) [9,10,11,12,13]. Serum accumulation of RAGE ligand AGEs initiates and drives the progression of diabetes [14,15,16]. The commonly identified clinical variant of AGEs demonstrates nonfluorescent modification of lysine, *N*-carboxymethyl-lysine (CML), on circulating proteins such as albumin [17]. Studies in animal models indicate that decreased CML may protect against diabetes, while human studies similarly show increased tissue/circulating CML concentrations to be predictive of diabetic complications [18,19].

RAGE is a membrane-bound protein that is structurally composed of three extracellular Ig-like domains, a single transmembrane helix, and a short intracellular domain [20]. Ligand binding to RAGE activates several pathways, including mitogen-activated protein kinases (MAPKs) and nuclear factor (NF)-κB, promoting inflammatory gene transcription, cell migration, and cell adhesion [21,22]. Under physiological conditions, RAGE is predominantly expressed in the lungs; however, RAGE levels rise when induced by ligands accumulated at injured sites in patients with diabetes [23,24]. A soluble (s) form of RAGE (sRAGE) is also present in the circulation and consists of shed membrane-bound RAGE ectodomain and secreted endogenous RAGE (esRAGE) [25,26]. Shed and esRAGE can be detected in the plasma but do not transduce signal upon ligand binding [25,26,27]. It has been postulated that sRAGE functions as a decoy molecule to bind excess RAGE ligand and, therefore, serve a protective role [25,26,27]. However, it is equally possible that increased sRAGE production is the result of reduced cell surface receptor expression, limiting the degree of cellular activation. Whether levels of sRAGE are associated with a better or worse outcome may, in part reflect these two roles. The production of soluble receptors, as a general concept, is regarded as a common feature of cytokine biology with implications for inflammatory disease progression and therapy. Maintaining high levels of circulating sRAGE isoforms can be advantageous for the host. In a rat model of type 1 diabetes, the administration of anti-RAGE antibodies or sRAGE blocked the increased vascular permeability [28]. In atherosclerosis-prone apoE-null mice made diabetic with streptozotocin sRAGE treatment markedly reduced the size and complexity of atherosclerotic lesions as well as the accumulation of RAGE ligands AGEs and S100s in the vessel wall. These experiments have been replicated in TD2 models with similar results [29,30] and demonstrate the feasibility of RAGE inhibition as a means of avoiding diabetic complications.

Cross-sectional human population studies report that circulating sRAGE is a potential biomarker of several pathologies, although conflicting data exists in the context of diabetes and cardiovascular diseases [31]. Decreased levels of circulating sRAGE have been reported in patients with hypertension, metabolic syndrome, coronary artery disease, T1D, T2D, and obesity, suggesting that sRAGE is an early predictor of cardiovascular risk [32,33,34,35,36,37]. In T2D, lower plasma sRAGE has been inversely correlated with HbA_1c_ [38]. Interestingly, in an apparently healthy population, sRAGE levels declined with age and were inversely associated with body mass index (BMI) [38]. Alternatively, studies have also demonstrated no difference or elevated total sRAGE levels in T1D and T2D compared with BMI-matched controls with no relationship to necessary measures of insulin sensitivity [39,40,41]. Furthermore, in T1D, increased sRAGE was associated with increased all-cause and cardiovascular mortality, potentially reflecting the activation and production of RAGE in the context of accelerated vascular disease [42]. The divergent data regarding circulating sRAGE concentrations in patients with diabetes are likely a consequence of the applied study designs.

These findings identify sRAGE to be a marker of diabetic complications. However, none of these studies address a key gap in our current knowledge—quantifying RAGE ligand levels, specifically the S100 proteins, in parallel with plasma sRAGE expression. Since diabetes may differentially impact the mechanisms controlling RAGE ligand generation and clearance, determining sRAGE levels in parallel with circulating RAGE ligands could be useful for assessing diabetic risk factors. In this study, we aimed to quantify total sRAGE, the RAGE ligands AGEs, S100B, S100A1 and S100A6 in a young, lean, healthy human reference group and middle-aged individuals stratified according to glucose tolerance status (GTS). We hypothesized that impaired glucose tolerance (IGT) and T2D reduces circulating sRAGE in comparison to a lean, healthy reference group. To validate our findings, we used a rodent abdominal aortic vascular smooth muscle cell (ASMC) culture model to determine whether circulating concentration of sRAGE acts as a decoy for cytoprotection against high glucose and/or RAGE ligand inflammatory/apoptotic actions.

## 2. Results

### 2.1. Anthropometric and Metabolic Characteristics

Table 1 presents the basic characteristics of the study population stratified by glucose tolerance status. There was an equal proportion of males and females in all groups. NGT-A individuals had a younger age and a decreased BMI compared to NGT-B, IGT and T2D. Comparing NGT-B, IGT, and T2D, weight and BMI were highest in T2D. Individuals with IGT and T2D had higher triglycerides and lower HDL cholesterol compared to NGT-B. The liver enzyme alanine aminotransferase was decreased in the IGT and T2D groups compared with NGT-B. Individuals with T2D had higher fasting glucose and insulin compared to NGT-B. Compared to NGT-B, 2-h administration of glucose or insulin was higher in IGT and T2D.

### 2.2. Serum sRAGE, S100A1, S100A6, AGEs, S100B and FasL Levels Are Differentially Regulated with Age and Diabetes

**Figure 1:**
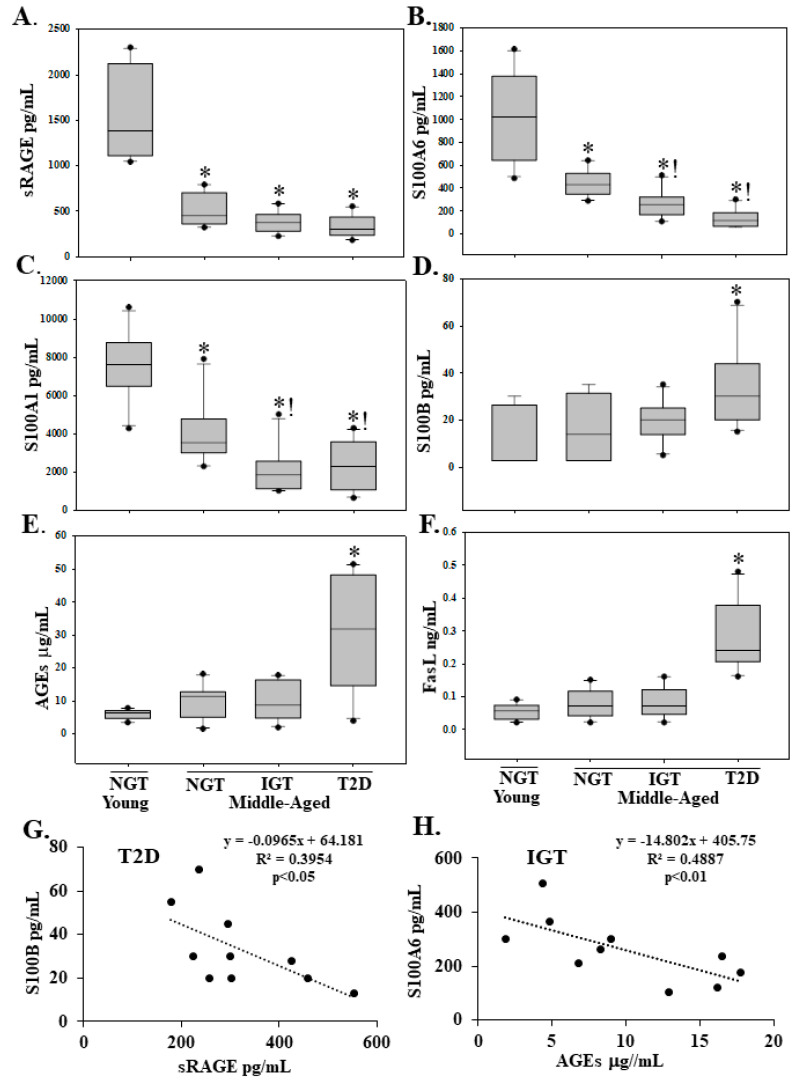
Plasma levels of sRAGE (**A**), the RAGE ligands S100A6 (**B**), S100A1 (**C**), S100B (**D**), AGEs (**E**) and the death receptor ligand FasL (**F**) in young individuals with normal glucose tolerance (NGT) and middle-aged with NGT, impaired GT, or Type II diabetics (T2D). Plasma levels as measured by enzyme-linked immunosorbent assay (ELISA) are presented as box plots showing median and interquartile range. * *p* < 0.05 vs. NGT-young; ! *p* < 0.05 vs. NGT-middle-aged. Negative correlations between S100B and sRAGE in type II diabetics (T2D) (**G**), and S100A6 and AGEs in individuals with impaired glucose tolerance (IGT) (**H**). *n* = 10.

The plasma level of the decoy sRAGE, that bind excess RAGE ligands, was decreased in middle-aged individuals with NGT-B, IGT and T2D compared to younger individuals with NGT-A (Figure 1A). Compared to NGT-A individuals, the plasma level of the RAGE ligands S100A6 and S100A1 was decreased in NGT-B and a further reduction was observed in IGT and T2D (Figure 1B,C). The death receptor pathway ligand FasL and the RAGE ligands AGEs and S100B were increased in T2D (Figure 1D–F). Strong negative correlations were observed between S100B and the decoy sRAGE (r = 0.629) and between S100A6 and AGEs (r = 0.699) (Figure 1G,H).

### 2.3. RAGE Ligands (AGES, S100B, S100A1, S100A6) and sRAGE Differentially Regulate Parameters of Apoptosis (BAX/BCL2 Ratio-Mitochondrial Pathway; Fas Ligand, Fas Receptor-Death Receptor Pathway) and Markers of Inflammation (TNF-α) in ASMC

**Figure 2:**
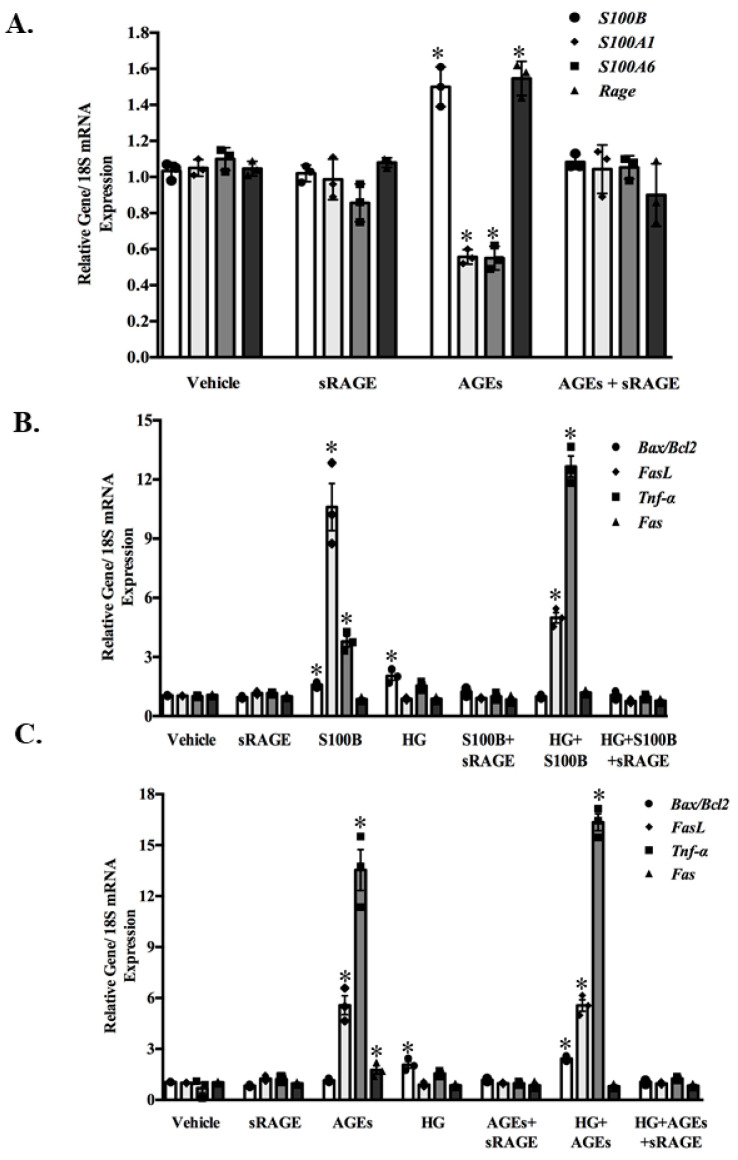
High glucose (HG), AGEs and sRAGE differentially regulate ligands of RAGE (**A**) and parameters of apoptosis (BAX/BCL2 ratio-mitochondrial pathway; FasL, Fas receptor and marker of inflammation (TNF-α) (**B**,**C**). Cultured adult rodent abdominal aortic smooth muscle cells were exposed to vehicle (PBS), sRAGE (1.5 ng/mL), high glucose (4.5 g/L), AGEs (50 μg/mL), or S100B (100 pg/mL) alone or in combination for 24 h. Gene mRNA expression was analyzed by real-time quantitative RT-PCR. All values are mean ± SEM of the ratio gene/18S relative to the vehicle. * *p* < 0.05 vs. vehicle. *n* = 6.

In cultured adult rodent abdominal ASMC, 24 h exposure to AGEs at a concentration (50 μg/mL) seen in the plasma of T2D increased the expression of S100B and RAGE and reduced S100A1 and S100A6 expression (Figure 2A). The addition of sRAGE at a concentration (1.5 ng/mL) observed in the plasma young NGT-A individuals to AGEs reduced the expression of S100B and RAGE and increased the expression of S100A1 and S100A6 to basal (vehicle) levels (Figure 2A). Treatment of ASMC with S100B at a concentration (100 pg/mL) seen in T2D increased the expression of the apoptotic gene ratio BAX/BCL2 reflective of mitochondrial apoptosis, the death receptor ligand FasL and the inflammation marker TNF-α (Figure 2B). Treatment of ASMC with AGEs (50 μg/mL), increased the expression of FasL, its receptor Fas and TNF-α (Figure 2C). Interestingly, the addition of HG (4.5 g/L) [43] to ASMC increased only the expression of the apoptotic ratio BAX/BCL2 (Figure 2B). HG in combination with S100B increased the expression of FasL and TNF-α, whereas HG in combination with AGEs increased the expression of the BAX/BCL2 ratio, FasL and TNF-α (Figure 2C). The addition of sRAGE to either S100B, AGEs, or HG alone or in combination abolished the observed increases in the apoptotic and inflammatory markers (Figure 2B,C).

**Figure 3:**
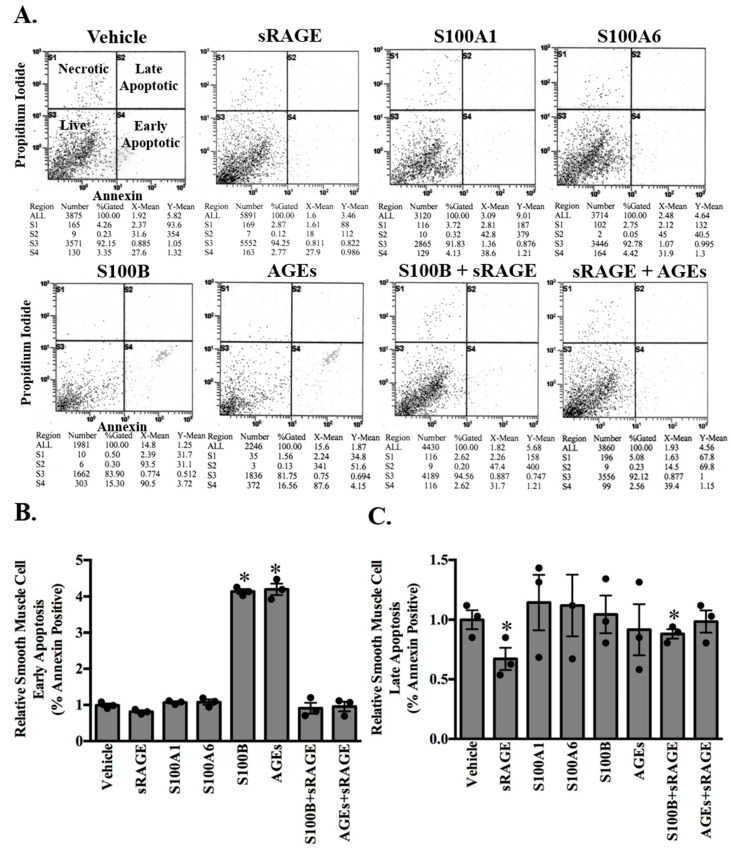
sRAGE inhibits smooth muscle cell apoptosis induced by either S100B or AGEs. Cultured adult rodent abdominal aortic smooth muscle cells were exposed to vehicle (PBS), sRAGE (1.5 ng/mL), S100A1 (10 ng/mL), S100A6 (1.5 ng/mL), AGEs (50 μg/mL) or S100B (100 pg/mL) alone or in combination with sRAGE (S100B, AGEs) for 24 h. Representative images of apoptosis as determined by flow cytometric analysis using the FITC Annexin V-Apoptosis kit are shown (**A**). Values are mean ± SEM of % Annexin positive cells relative to vehicle in early (**B**) and late (**C**) apoptosis. * *p* < 0.05 vs. vehicle. *n* = 6.

To determine whether the RAGE ligands AGEs, S100B, S100A1, and S100A6 directly induce ASMC early and late apoptosis, ASMC were treated for 24 h with either vehicle (PBS), sRAGE (1.5 ng/mL), S100B (100 pg/mL), AGEs (50 μg/mL), S100A6 (1.5 ng/mL-concentration observed in the plasma of young NGT-A), or S100A1 (10 ng/mL-concentration observed in young NGT-A) (Figure 3A–C). Treatment with only the RAGE ligands S100B and AGEs increased early apoptosis as quantified by the proportion of Annexin V positive cells, responses abrogated in the presence of sRAGE (Figure 3A,B). Interestingly, late apoptotic events were not observed with any of the RAGE ligands (Figure 3A,C). Apoptosis is mediated by activation of the caspase cascades [44]. 

**Figure 4:**
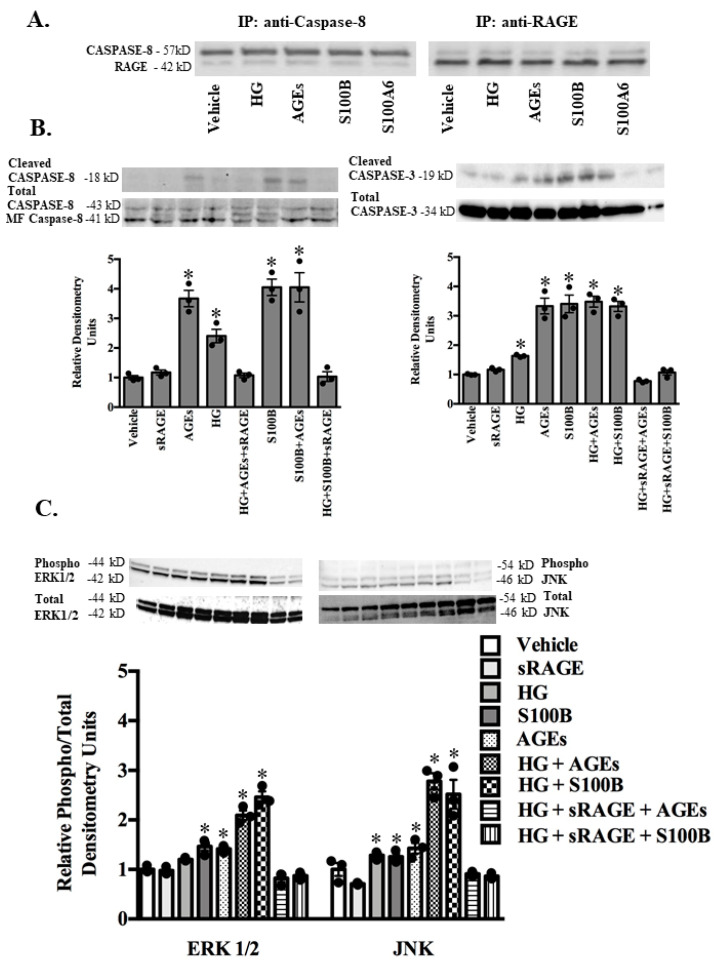
RAGE ligands regulate caspase-3, -8 activation and MAPK (ERK1/2, JNK) signaling. Cultured adult rodent abdominal aortic smooth muscle cells were exposed to vehicle (PBS), sRAGE (1.5 ng/mL), high glucose (HG) (4.5 g/mL), AGEs (50 μg/mL) or S100B (100 pg/mL) alone or in combination with sRAGE for 24 h. Representative immunoprecipitation assay showing an association of RAGE and caspase-8 following treatment with vehicle (PBS), high glucose (HG), AGEs or S100B (**A**). Representative Western blots show cleaved, mid-form (MF) and total caspase-8, cleaved and total caspase-3 (**B**). Densitometry values are mean + SEM of cleaved/total relative to the vehicle. Representative Western blot shows phospho and total ERK1/2 and JNK (**C**). Densitometry values are mean + SEM relative to vehicle (PBS). * *p* < 0.05 vs. vehicle. *n* = 3.

To define the cellular mechanisms by which RAGE ligation leads to apoptosis, we evaluated the cleavage of the initiator death receptor pathway caspase-8 and the executioner mitochondria-dependent caspase-3. A co-immunoprecipitation strategy followed by Western blot analysis demonstrated heterodimerization of caspase-8 and RAGE in ASMC cell lysates treated with vehicle (PBS), HG, AGEs, S100B, or S100A6 (Figure 4A). As represented in Western blots and quantified by densitometry, exposure of ASMC to either HG, AGEs or S100B alone or in combination resulted in increased cleavage of both caspase-8 and -3, responses abrogated in the presence of sRAGE (Figure 4B).

### 2.4. RAGE-Ligand Binding Induces the MAPK Signaling Pathway

The MAPK signaling pathway plays an important role in diabetic vascular damage [17,22]. Therefore, we examined the effect of RAGE ligands on the activation of the MAPK signaling pathway in AVSM. AVSM were treated with either HG, the proapoptotic RAGE ligands AGEs or S100B alone, or in combination with sRAGE, and phosphorylation of ERK1/2 and JNK was determined by resolving cell lysates on Western blots (Figure 4C). Densitometry measurements demonstrated treatment with HG, AGEs, or S100B alone or in combination increased the phosphorylation of ERK1/2 and JNK (Figure 4C). The addition of sRAGE abrogated ERK1/2 and JNK phosphorylation (Figure 4C).

## 3. Discussion

To our knowledge, the present study is the first to report circulating concentrations of sRAGE with the RAGE ligands AGEs, S100B, S100A1 and S100A6 in the context of young, healthy human individuals and middle-aged individuals who are either healthy, have impaired glucose tolerance, or T2D. Our primary findings show young individuals present the highest concentration of sRAGE, anti-apoptotic S100A1, and S100A6 and the lowest concentration of pro-apoptotic AGEs and S100B when compared to middle-aged individuals with NGT, IGT or T2D. These findings are consistent with previous reports showing lower sRAGE expression in patients with obesity and impaired glucose tolerance [33,34,35,36,37]. Importantly, in ASMC, we demonstrate that T2D circulating concentrations of AGEs and S100B induce signaling pathways resulting in inflammatory and apoptosis responses that are attenuated in the presence of sRAGE at a concentration seen in the plasma of young NGT individuals.

Our data are, admittedly, limited by the number of individuals per group and not being age-matched across all groups, but do show that chronological age plays an essential role in sRAGE concentration. Secondly, we acknowledge that this study was completed at a single site with one demographic population (Greek). Finally, our data is limited by the measurement of only total sRAGE using the R&D ELISA kit. Total sRAGE levels decrease with advancing age. Total sRAGE consists of two circulating soluble isoforms: esRAGE derived from alternative splicing and cRAGE generated by proteolytic cleavage of RAGE by ADAM10 and/or membrane-bound RAGE proteolysis (MMPs). esRAGE does not vary during aging and eventually represents the predominant circulating sRAGE isoform in the elderly [45]. Conversely, cRAGE is the most abundant circulating sRAGE in young individuals and decreases with age dropping to dismal levels in the elderly [45]. In our study, the high circulating sRAGE concentration in young individuals may be reflective of the cRAGE isoform that decreases with age.

Interestingly, in middle-aged individuals, circulating sRAGE in states of impaired glucose tolerance and T2D remains comparable to NGT. Conflicting results have been reported regarding circulating sRAGE and T2D [32,33,34,35,36,37]. In contrast to our data, others have reported higher circulating sRAGE levels in type 2 diabetic patients compared to nondiabetic subjects and positively associated with the presence of coronary artery disease in diabetes [39,40,41]. The endogenous sRAGE level may be elevated in diabetes as a counter-system against endothelial cell damage and could reflect enhanced RAGE expression in the diabetic vasculature. Alternatively, attenuated total sRAGE has been independently reported with obesity, prediabetes, and T2D, and low total sRAGE was associated with greater risk of developing T2D and cardiovascular mortality in nondiabetic individuals [31,32,33,34,35,36,37]. Differences in the makeup of the T2D subjects, along with the type of ELISA kit used to measure sRAGE may account for the discrepant results.

Our study also reveals that aging also decreases the concentration of the cytoprotective RAGE ligands S100A1 and S100A6 with a further decrease in states of IGT and T2D whereas pro-apoptotic/inflammatory circulating AGEs and S100B accumulate in T2D. Additionally, inverse correlations between sRAGE and S100B in T2D, and S100A6 and AGEs in IGT characterize middle age states of IGT and T2D by a shift from cytoprotective (S100A1, S100A6) to pro-apoptotic/pro-inflammatory (S100B, AGEs) RAGE ligands. However, the juxtaposition of the T2D phenotype against a young, healthy phenotype demonstrates the degree to which circulating sRAGE, S100A1, S100A6, AGES, and S100B in states of IGT, and advanced age deviate from optimum health. We have demonstrated here that dysregulation of sRAGE and ligands of RAGE is associated with different phenotypes, and therefore are likely under parallel regulation. The progressive decrease of sRAGE and cytoprotective S100A1 and S100A6 with aging may result in the accumulation of AGEs and S100B, fueling signaling pathways favoring the diabetic state.

It is well established that the binding of RAGE with ligands drives a cascade of signaling that converges at NF-κB, promoting inflammatory and apoptotic processes [17,22]. RAGE triggers the inflammatory intracellular signal transduction pathways of a variety of cells, promoting the secretion of pro-inflammatory cytokines such as TNF-α, leading to acute inflammation and cellular dysfunction. Since vascular SMC plays pivotal roles in the occurrence of diabetic complications such as atherosclerosis, we investigated the role of sRAGE in reducing the harmful effects of high-glucose (HG) or the RAGE ligands AGEs and S100B in cultured ASMC, and identified associated molecular mechanisms. RAGE engagement by a myriad of ligands is linked to an array of signaling pathways. These include the activation of NF-κB, MAPKs, PI3K/Akt, Rho GTPases, Jak/STAT, and Src family kinases, among others [17,22]. This variety of reported RAGE signal transduction pathways is quite extraordinary. However, the diverse nature of the RAGE ligands, and possibly their contaminating elements, coupled with the broad expression pattern of RAGE may account for such an assortment of signals. RAGE is known to transduce differential signals depending on the ligand concentration. In the picomolar range, S100B behaves like a neurotrophin protecting neuronal cells against neurotoxic stimuli through stimulation of ERK1/2 and NF-κB-mediated upregulation of the anti-apoptotic mediators, whereas at the nano- to micromolar doses, it is neurotoxic through excess MAPK stimulation and ROS production [12]. S100B in the plasma of young and middle-age individuals is found in picomolar amounts and increases in T2D patients. Exposure of ASMC to T2D circulating concentrations of AGEs and S100B provokes inflammatory and apoptotic signaling resulting in increased expression of TNF-α, the apoptotic BAX/BCL2 ratio, and the death receptor pathway component FasL. AGEs/S100B-induced apoptosis is largely dependent on the effector caspase, caspase-3, which is activated through both the extrinsic cell death pathway (caspase-8-dependent) and mitochondrial intrinsic cell death (BAX/BCL2 dependent) pathway. Our results align with reports in the literature that indicate caspase-8 regulates not only cell death but also inflammation signaling [44,46,47]. The concurrent pro-inflammatory and apoptotic events induced by AGEs and S100B are inhibited in the presence of the decoy receptor sRAGE at a concentration seen in young NGT individuals. Thus, with advancing age, the decline in sRAGE and cytoprotective S100A1 and S100A6 is permissive for AGEs/S100B RAGE-dependent inflammatory/apoptotic events. In SMCs, MAPK appears to be the principal kinases activated by RAGE ligands. Indeed, ERK1/2 is a direct RAGE-binding partner [48]. AGEs or S100B via RAGE signaling elevate the level of ERK and JNK phosphorylation. Activation of both JNK and ERK, components of the intracellular downstream signal transduction pathway of RAGE, is associated with inflammation and/or apoptosis, which is consistent with our results.

RAGE is a pattern-recognition receptor that predominately binds to endogenous molecules that are generated or released during cellular, physiological, or pathological stresses [9,10,11,12,13]. It has been proposed that tissue damage releases endogenous DAMPs, which are quintessential for initiating the restoration of tissue homeostasis. DAMP molecules include the RAGE ligands AGEs and members of the S100 family. Our data suggest that AGEs and S100B released from damaged cells in response to the diabetic state stimulate inflammatory and apoptotic responses in ASMC by oligomerizing with the RAGE receptor. Importantly, the use of ASMC from healthy rats may be sufficient in evaluating RAGE ligand-induced apoptotic and inflammatory responses but, it is not equivalent to ASMC from middle-aged patients with T2D.

## 4. Materials and Methods

### 4.1. Study Populations

A large number of middle-aged (36–65 years) Greek diabetic patients with an initial diagnosis different from insulin-dependent diabetes mellitus (IDDM) (type 1 diabetes) were evaluated at the outpatient Diabetic Clinic of Attikon University General Hospital in Athens, Greece, between June 2009 and May 2012 after giving their informed consent. Patients with infection, liver disease, or pregnancy were excluded from the study. Upon enrollment into the study, height and weight measurements were collected from each patient. After overnight fasting, data from a 75-g oral glucose tolerance test (OGTT) was collected from each patient. In parallel, blood samples were obtained through peripheral venipuncture at 0 and 120 min to measure plasma glucose, serum insulin, and serum C-peptide concentrations. Patients with a known history of diabetes or a fasting plasma glucose ≥126 mg/dL did not undergo an OGTT and were excluded from the study. A total of 30 patients were enrolled in the study and classified according to the criteria by the American Diabetes Association into 10 individuals with normal glucose tolerance (NGT), 10 with impaired glucose tolerance (IGT) and 10 with type 2 diabetes (T2D). Ten healthy control individuals (<35 years of age) were used as a reference group. All subjects had no family history of diabetes mellitus, demonstrated normal blood glucose levels, and provided informed consent.

### 4.2. Ethics Statement

The institutional review board of “Attikon” University General Hospital approved this study during the 19th meeting 11 March 2009. All patients gave their written informed consent.

### 4.3. Laboratory Analyses

Serum cholesterol (total, low-density lipoprotein (LDL), and high-density lipoprotein (HDL)), triglycerides, aspartate transaminase (AST), alanine aminotransferase (ALT), and gamma glutamyl transferase (γ-GT) were assessed using a Cobas 8000 analyzer (Roche, Basel, Switzerland). Plasma glucose was measured using the glucose oxidase method (YSI, Yellow Springs Instruments, Yellow Springs, CO, USA). Serum C-peptide and insulin levels were quantified using the respective radio-immunoassays from Millipore (St. Charles, MO, USA).

### 4.4. Blood Sampling and Laboratory Methods

Total plasma sRAGE in humans was determined by a commercial enzyme-linked immunosorbent assay (ELISA) (DY1145, R&D Systems, Minneapolis, MN, USA). S100B (DY18020-5, R&D Systems, Minneapolis, MN), AGEs (STA-817, Cell Biolabs, San Diego, CA, USA), and FasL (DY126, R&D Systems, Minneapolis, MN, USA) levels were determined by enzyme immunometric assay according to manufacturer instructions. For the human S100A1 ELISA, a 96-well plate was coated with a rabbit anti-S100A1 antibody (ab11428, Abcam, Cambridge, MA, USA) and incubated overnight at 4 °C temperature. Recombinant S100A1 (#10-663-45642, Genway, San Diego, CA, USA) serving as a standard, and experimental samples were added and incubated overnight at 4 °C. Sheep anti-human S100A1 (dilution of 1/5000, AF4476, R&D systems, Minneapolis, MN, USA) was added, followed by incubation for 3 h at room temperature. Donkey anti-sheep antibody (sc-2473, Santa Cruz, Santa Cruz, CA, USA) was added and further incubated for 2 h at room temperature. TMB substrate (ADI-80091, Enzo Life Sciences, Farmingdale, NY, USA) was added followed by incubation for 20 min at room temperature in the dark. The reaction was stopped by adding stop solution, and the color was read at 450 nm with a correction at 570 nm. For the S100A6 ELISA, a 96 well plate was coated with sheep anti-S100A6 (AF4584, R&D Systems, Minneapolis, MN, USA) overnight at 4 °C then washed and blocked overnight. Aliquots of recombinant S100A6 (Swant, Marly, Switzerland) serving as a standard and experimental samples were added and incubated overnight at 4 °C. Chicken anti-S100A6 (1:500, GW22597F, Millipore Sigma, St. Louis, MO, USA) solution was added followed by a 3 h incubation at room temperature. Goat anti-chicken HRP (ab6877, Abcam, Cambridge, MA, USA) was added and the samples were incubated for an additional 2 h. TMB substrate was added followed by a 20 min incubation for 20 min at room temperature. The reaction was stopped by the addition of stop solution and the color was read at 450 nm with a correction at 570 nm.

### 4.5. Cell Culture

Primary cultures of vascular smooth muscle cells (SMC) were initiated by enzymatic dissociation from the abdominal aortas (A) of eight-week-old Sprague Dawley rats, as previously described [49]. Cultured ASMC were exposed to vehicle (PBS), sRAGE (1.5 ng/mL; PRO-601, Prospec Protein Specialists, East Brunswick, NJ, USA), high glucose (4.5 g/L), AGEs (50 μg/mL; ab51995, Abcam, Cambridge, MA, USA), S100B (100 pg/mL; 1820-SA-050, R&D Biosystems, Minneapolis, MN, USA), S100A1 (10 ng/mL; #10-663-45642, Genway, San Diego, CA, USA), or S100A6 (1.5 ng/mL; Swant, Marly, Switzerland) alone or in combination for 24 h.

### 4.6. Immunoprecipitation and Western Blot Analysis

Cell lysates were prepared from frozen powder, and immunoprecipitation studies and Western blotting were performed as described previously [43,46,50]. In brief, for immunoprecipitation, 0.5 mg of cell lysates were incubated with the primary antibody RAGE (5 mL, ab216329, Abcam, Cambridge, MA, USA) or caspase-8 antibody (5 μL, #9746, Cell Signaling Technology, Danvers, MA, USA) overnight on a rotating shaker. Following washing, the mixture was incubated with A/G Agarose beads suspension (Santa Cruz, Santa Cruz, CA, USA) for 1 h at room temperature. Following washing (3–5 times), the pellet was re-suspended in SDS loading buffer and boiled for 5 min. Supernatants were loaded and gel electrophoresis was performed. Blots were stripped and probed with the appropriate antibodies. Western blot analysis was performed with phosphorylated (phospho)-extracellular related kinase (ERK)1/2, total ERK1/2 (#9911, Cell Signaling Technology, Danvers, MA, USA), phospho-c-Jun *N*-terminal kinase (JNK), total JNK (#9250, Cell Signaling Technology, Danvers, MA, USA), cleaved and total caspase-3 (cleaved AB3623, total AB1899, Millipore Sigma, St. Louis, MO, USA) and caspase-8 (#9746, Cell Signaling Technology, Danvers, MA, USA) antibodies.

### 4.7. Real-Time Quantitative RT-PCR

RNA was extracted from primary cultures of abdominal aortic vascular smooth muscle cells using Trizol (Sigma, St. Louis, MO, USA) and reverse transcribed using QuantiTeck kit (Qiagen, Alameda, CA, USA). Real time quantitative (RT)-PCR was performed according to the instructions of the manufacturer (Qiagen) and as previously described [43,50]. In brief, 1 μL of gene-specific 10 μM PCR primer pair stock of gene of interest (rodent S100B, S100A1, S100A6, Rage, B-cell lymphoma (BCL)2, Bcl2-associated X (BAX), Fas ligand (L), Tumor necrosis factor (TNF)-α, Fas receptor, 18S-proprietary sequences) (Qiagen, Waltham, MA, USA) and 1 μL of cDNA (template) underwent a two-step cycling program, 40 cycles, 10 min at 95 °C, 15 s at 95 °C, and 1 min at 60 °C. In separate experiments, the threshold cycle (C_t_) values for the housekeeping gene 18S and the gene of interest in each sample were determined. For each gene of interest, the mean fold-change is shown relative to vehicle/control.

### 4.8. Measurements of Apoptosis

Cellular apoptosis was determined by flow cytometric analysis using the FITC Annexin V-Apoptosis detection kit (BD Bioscience, San Diego, CA, USA). Caspase-3 activity was measured using Enzchek^TM^ caspase-3 Assay kit *#*2 (Molecular Probes Inc., Eugene, OR, USA).

### 4.9. Statistical Analysis

The study sample characteristics are given as percentages for categorical variables, mean and standard error of the mean (SEM) for continuous variables with normal distribution, and median (25th and 75th percentiles) for continuous variables without normal distribution. The Kolmogorov-Smirnov test was used to test for normal distribution of data. Group comparisons for these variables were performed using Fisher’s exact test, t-test, or ANOVA with Dunnett’s multiple comparison test (for two or more groups) and Kruskal-Wallis test with Dunn’s multiple comparison test for more than two groups, respectively. Correlations were assessed as partial Spearman correlation coefficients adjusting for the variables indicated. *p* values lower than 0.05 were considered statistically significant.

## 5. Conclusions

In conclusion, younger individuals exhibit high circulating levels of the decoy sRAGE. In middle-aged individuals, particularly those with T2D, decreased plasma sRAGE may limit decoy and exogenous trapping of deleterious RAGE ligands AGEs and S100B, increasing the risk for diabetic complications. Our in vitro results with ASMC support pro-inflammatory and proapoptotic effects of AGEs and S100B at concentrations observed in the plasma of T2D. Taken together, these studies suggest that clinically measuring sRAGE and RAGE associated ligands, namely AGEs and S100 proteins, may serve as a feasible diagnostic and/or prognostic marker to assess the risk of developing diabetes and diabetes-induced complications as individuals age. Importantly, prospective clinical trials will have to define the impact of sRAGE and RAGE ligands as biomarkers of cardiovascular risk in T2D.

## Figures and Tables

**Table 1:** Patient Characteristics. NGT-normal glucose tolerance; IGT-impaired glucose tolerance; T2D-type 2 diabetes; Weight; BMI-body mass index; HDL-high density lipoprotein; LDL-low density lipoprotein; AST-aspartate aminotransferase; ALT-alanine aminotransferase; GT-glutamy-ltransferase. ^#^ *p* < 0.05 vs. control (young), * *p* < 0.05 vs. control (aged), ^!^ *p* < 0.05 vs. IGT.

Variable	NGT-A	NGT-B	IGT	T2D
	Young	Middle-Aged	Middle-Aged	Middle-Aged
n(%men)	10 (50)	10 (50)	10 (50)	10 (70)
Age, years	32.1 ± 1.5	59.6 ± 1.2 ^#^	59.8 ± 1.6 ^#^	59.8 ± 1.6 ^#^
Weight, kg	73.9 ± 4.7	75.1 ± 2.9	80.5 ± 4.3	91.5 ± 3.1 ^#^
BMI, kg/m^2^	24.0 ± 0.9	27.9 ± 0.7 ^#^	28.1 ± 0.66 ^#^	31.8 ± 0.9 *^#^
**Serum Lipids**				
Triglycerides, mg/dL		114 ± 16.1	140 ± 20.2 *	140 ± 21.8 *
Total Cholesterol, mg/dL		229 ± 11.3	215 ± 13.1	217 ± 13.6
HDL Cholesterol, mg/dL		59.7 ± 4.5	51.8 ± 4.7	44.2 ± 3.9 *
LDL, Cholesterol, mg/dL		138 ± 8.9	129 ± 11.9	140 ± 9.7
**Liver Enzymes**				
AST, U/L		32 (17, 62)	30 (19, 47)	28 (12, 54)
ALT, U/L		54 (15, 124)	43 (27, 80) *	44 (26, 65) *
g-GT, U/L		60 (12, 138)	57 (12, 174)	63 (6, 165)
**Measures of Glycemia**				
Fasting glucose, mg/dL		86.1 ± 3.3	82.5 ± 3.9	108.2 ± 9.3 *
2-h glucose, mg/dL		113 ± 4.3	154.8 ± 4.7 *	251.9 ± 15.5 *^!^
Fasting insulin, μU/mL		10.8 ± 1.4	11.3 ± 1.3	15.9 ± 2.8 *
2-h insulin μU/mL		94.3 ± 16.9	162.2 ± 37.4 *	150.9 ± 22.1 *

## References

[1] Atkinson M.A., Eisenbarth G.S., Michels A.W. (2014). Type 1 diabetes. Lancet.

[2] Herman W.H., Zimmet P. (2012). Type 2 diabetes: An epidemic requiring global attention and urgent action. Diabetes Care.

[3] Rhodes E.T., Prosser L.A., Hoerger T.J., Lieu T., Ludwig D.S., Laffel L.M. (2012). Estimated morbidity and mortality in adolescents and young adults diagnosed with type 2 diabetes mellitus. Diabet. Med..

[4] Coughlan M.T., Yap F.Y.T., Tong D.C.K., Andrikopoulos S., Gasser A., Thallas-Bonke V., Webster D.E., Miyazaki J.I., Kay T.W., Slattery R.M. (2011). Advanced glycation end products are direct modulators of beta-cell function. Diabetes.

[5] Yamagishi S., Imaizumi T. (2005). Diabetic vascular complications: Pathophysiology, biochemical basis and potential therapeutic strategy. Curr. Pharm. Des..

[6] Sourris K.C., Forbes J.M. (2009). Interactions between advanced glycation end-products (AGE) and their receptors in the development and progression of diabetic nephropathy–are these receptors valid therapeutic targets. Curr. Drug Targets.

[7] Daffu G., del Pozo C.H., O’Shea K.M., Ananthakrishnan R., Ramasamy R., Schmidt A.M. (2013). Radical roles for RAGE in the pathogenesis of oxidative stress in cardiovascular diseases and beyond. Int. J. Mol. Sci..

[8] Fukami K., Yamagishi S., Okuda S. (2014). Role of AGEs-RAGE system in cardiovascular disease. Curr. Pharm. Des..

[9] Yan S.F., Ramasamy R., Schmidt A.M. (2010). The RAGE axis: A fundamental axis signaling danger to the vulnerable vasculature. Circ. Res..

[10] Yamagishi S. (2011). Role of advanced glycation end products (AGEs) and receptor for AGEs (RAGE) in vascular damage in diabetes. Exp. Gerontol..

[11] Ramasamy R., Yan S.F., Schmidt A.M. (2012). The diverse ligand repertoire of the receptor for advanced glycation endproducts & pathways to the complications of diabetes. Vascul. Pharmacol..

[12] Donato R., Cannon B.R., Sorci G., Riuzzi F., Hsu K., Weber D.J., Geczy C.L. (2013). Functions of S100 proteins. Curr. Mol. Med..

[13] Bianchi M.E. (2007). DAMPs, PAMPs and alarmins: All we need to know about danger. J. Leukoc. Biol..

[14] Bierhaus A., Hofmann M.A., Ziegler R., Nawroth P.P. (1998). AGEs and their interaction with AGE-receptors in vascular disease and diabetes mellitus. I. The AGE concept. Cardiovasc. Res..

[15] Nathan D.M., Genuth S., Lachin J., Cleary P., Crofford O., Davis M., Rand L., Siebert C., The Diabetes Control and Complications Trial Research Group (1993). The effect of intensive treatment of diabetes on the development and progression of long-term complications in insulin dependent diabetes mellitus. N. Engl. J. Med..

[16] Stratton I.M., Adler A.I., Neil H.A., Matthews D.R., Manley S.E., Cull C.A., Hadden D., Turner R.C., Holman R.R. (2000). Association of glycaemia with macrovascular and microvascular complications of type 2 diabetes (UKPDS 35): Prospective observational study. BMJ.

[17] Kislinger T.K., Fu C.B., Huber B., Qu W., Taguchi A., Yan S.D., Hofmann M., Yan S.F., Pischetsrieder M., Stern D. (1999). *N*^ε^-(carboxymethyl)lysine adducts of proteins are ligands for advanced glycation end products that activate cell signaling pathways and modulate gene expression. J. Biol. Chem..

[18] Beisswenger P.J., Makita Z., Curphey T.J., Moore L.L., Jean S., Brinck-Johnsen T., Bucala R., Vlassara H. (1995). Formation of immunochemical advanced glycosylation end products precedes and correlates with early manifestations of renal and retinal disease in diabetes. Diabetes.

[19] Yanagisawa K., Makita Z., Shiroshita K., Ueda T., Fusegawa T., Kuwajima S., Takeuchi M., Koike T. (1998). Specific fluorescence assay for advanced glycation end products in blood and urine of diabetic patients. Metabolism.

[20] Fritz G. (2011). RAGE: A single receptor fits multiple ligands. Trends Biochem. Sci..

[21] Kierdorf K., Fritz G. (2013). RAGE regulation and signaling in inflammation and beyond. J. Leukoc. Biol..

[22] Rouhiainen A., Kuja-Panula J., Tumova S., Rauvala H. (2013). RAGE-mediated cell signaling. Methods Mol. Biol..

[23] Yao D., Brownlee M. (2010). Hyperglycemia-induced reactive oxygen species increase expression of the receptor for advanced glycation end products (RAGE) and RAGE ligands. Diabetes.

[24] Fehrenbach H., Kasper M., Tschernig T., Shearman M.S., Schuh D., Müller M. (1998). Receptor for advanced glycation end products (RAGE) exhibits highly differential cellular and subcellular localization in rat and human lung. Cell. Mol. Biol..

[25] Vazzana N., Santilli F., Cuccurullo C., Davì G. (2009). Soluble forms of RAGE in internal medicine. Intern. Emerg. Med..

[26] Raucci A., Cugusi S., Antonelli A., Barabino S.M., Monti L., Bierhaus A., Reiss K., Saftig P., Bianchi M.E. (2008). A soluble form of the receptor for advanced glycation endproducts (RAGE) is produced by proteolytic cleavage of the membrane-bound form by the sheddase a disintegrin and metalloprotease 10 (ADAM10). FASEB J..

[27] Yamagishi S., Matsui T. (2010). Soluble form of a receptor for advanced glycation end products (sRAGE) as a biomarker. Front. Biosci..

[28] Wautier J.L., Zoukourian C., Chappey O., Wautier M.P., Guillausseau P.J., Cao R., Hori O., Stern D., Schmidt A.M. (1996). Receptor-mediated endothelial cell dysfunction in diabetic vasculopathy. Soluble receptor for advanced glycation end products blocks hyperpermeability in diabetic rats. J. Clin. Investig..

[29] Park L., Raman K.G., Lee K.J., Lu Y., Ferran L.J., Chow W.S., Stern D., Schmidt A.M. (1998). Suppression of accelerated diabetic atherosclerosis by the soluble receptor for advanced glycation endproducts. Nat. Med..

[30] Wendt T., Harja E., Bucciarelli L., Qu W., Lu Y., Rong L.L., Jenkins D.G., Stein G., Schmidt A.M., Yan S.F. (2006). RAGE modulates vascular inflammation and atherosclerosis in a murine model of type 2 diabetes. Atherosclerosis.

[31] Bucciarelli L.G., Wendt T., Qu W., Lu Y., Lalla E., Rong L.L., Goova M.T., Moser B., Kislinger T., Lee D.C. (2002). RAGE blockade stabilizes established atherosclerosis in diabetic apolipoprotein E-null mice. Circulation.

[32] Prasad K. (2014). Low levels of serum soluble receptors for advanced glycation end products, biomarkers for disease state: Myth or reality. Int. J. Angiol..

[33] Falcone C., Emanuele E., D’Angelo A., Buzzi M.P., Belvito C., Cuccia M., Geroldi D. (2005). Plasma levels of soluble receptor for advanced glycation end products and coronary artery disease in nondiabetic men. Arterioscler. Thromb. Vasc. Biol..

[34] Lindsey J.B., de Lemos J.A., Cipollone F., Cipollone F., Ayers C.R., Rohatgi A., Morrow D.A., Khera A., McGuire D.K. (2009). Association between circulating soluble receptor for advanced glycation end products and atherosclerosis: Observations from the dallas heart study. Diabetes Care.

[35] Hudson B.I., Moon Y.P., Kalea A.Z., Khatri M., Marquez C., Schmidt A.M., Paik M.C., Yoshita M., Sacco R.L., De Carli C. (2011). Association of serum soluble receptor for advanced glycation end-products with subclinical cerebrovascular disease: The Northern Manhattan Study (NOMAS). Atherosclerosis.

[36] Selvin E.M., Halushka M.K., Rawlings A.M., Selvin E., Halushka M.K., Rawlings A.M., Hoogeveen R.C., Ballantyne C.M., Coresh J., Astor B.C. (2013). sRAGE and risk of diabetes, cardiovascular disease, and death. Diabetes.

[37] Colhoun H.M., Betteridge D.J., Durrington P., Hitman G., Neil A., Livingstone S., Menys V.C., Bao W., Demicco D.A., Preston G.M. (2011). Total soluble and endogenous secretory receptor for advanced glycation end products as predictive biomarkers of coronary heart disease risk in patients with type 2 diabetes: An analysis from the CARDS trial. Diabetes.

[38] Farhan S.S., Hussain S.A. (2019). Advanced glycation end products (AGEs) and their soluble receptors (sRAGE) as early predictors of reno-vascular complications in patients with uncontrolled type 2 diabetes mellitus. Diabetes Metab. Syndr..

[39] Fujisawa K., Katakami N., Kaneto H., Naka T., Takahara M., Sakamoto F., Irie Y., Miyashita M., Kubo F., Yasuda T. (2013). Circulating soluble RAGE as a predictive biomarker of cardiovascular event risk in patients with type 2 diabetes. Atherosclerosis.

[40] Tan K.C.B., Shiu S.W.M., Chow W.S., Leng L., Bucala R., Betteridge D.J. (2006). Association between serum levels of soluble receptor for advanced glycation end products and circulating advanced glycation end products in type 2 diabetes. Diabetologia.

[41] Challier M., Jacqueminet S., Benabdesselam O., Grimaldi A., Beaudeux J.L. (2005). Increased serum concentrations of soluble receptor for advanced glycation endproducts in patients with type 1 diabetes. Clin. Chem..

[42] Nin J.W., Jorsal A., Ferreira I., Schalkwijk C.G., Prins M.H., Parving H.H., Tarnow L., Rossing P., Stehouwer C.D.A. (2010). Higher plasma soluble Receptor for Advanced Glycation End Products (sRAGE) levels are associated with incident cardiovascular disease and all-cause mortality in type 1 diabetes: A 12-year follow-up study. Diabetes.

[43] Mohammadzadeh F., Tsoporis J.N., Izhar S., Desjardins J.F., Parker T.G. (2018). Deficiency of S100B confers resistance to experimental diabetes in mice. Exp. Cell Res..

[44] Van Opdenbosch N., Lamkanfi M. (2019). Caspases in cell death, inflammation, and disease. Immunity.

[45] Edwin R., Miranda E.R., Somal V.S., Blackburn B.K., Wang E., Farabi S., Karstoft K., Fealy C.E., Kashyap S., Kirwan J.P. (2017). Circulating soluble RAGE isoforms are attenuated in obese, impaired-glucose-tolerant individuals and are associated with the development of type 2 diabetes. Am. J. Physiol. Endocrinol. Metab..

[46] Gupta S., Lee C.M., Wang J.F., Parodo J., Jia S.H., Hu J., Marshall J.C. (2018). Heat-shock protein-90 prolongs septic neutrophil survival by protecting c-Src kinase and caspase-8 from proteasomal degradation. J. Leukoc. Biol..

[47] Maelfait J., Vercammen E., Janssens S., Schotte P., Haegman M., Magez S., Beyaert R. (2008). Stimulation of Toll-like receptor 3 and 4 induces interleukin-1beta maturation by caspase-8. J. Exp. Med..

[48] Ishihara K., Tsutsumi K., Kawane S., Nakajima M., Kasaoka T. (2003). The receptor for advanced glycation end-products (RAGE) directly binds to ERK by a D-domain-like docking site. FEBS Lett..

[49] Tsoporis J.N., Overgaard C.B., Izhar S., Parker T.G. (2009). S100B modulates the hemodynamic response to norepinephrine stimulation. Am. J. Hypertens..

[50] Mohammadzadeh F., Desjardins J.F., Tsoporis J.N., Proteau G., Leong-Poi H., Parker T.G. (2013). S100B: Role in cardiac remodeling and function following myocardial infarction in diabetes. Life Sci..

